# Structure and Function of Rhizosphere Soil and Root Endophytic Microbial Communities Associated With Root Rot of *Panax notoginseng*

**DOI:** 10.3389/fpls.2021.752683

**Published:** 2022-01-05

**Authors:** Panpan Wang, Lifang Yang, Jialing Sun, Ye Yang, Yuan Qu, Chengxiao Wang, Diqiu Liu, Luqi Huang, Xiuming Cui, Yuan Liu

**Affiliations:** ^1^Faculty of Life Science and Technology, Kunming University of Science and Technology, Kunming, China; ^2^Yunnan Provincial Key Laboratory of Panax notoginseng, Kunming, China; ^3^Key Laboratory of Panax notoginseng Resources Sustainable Development and Utilization of State Administration of Traditional Chinese Medicine, Kunming, China; ^4^Kunming Key Laboratory of Sustainable Development and Utilization of Famous-Region Drug, Kunming, China; ^5^National Resource Center for Chinese Materia Medica, China Academy of Chinese Medical Sciences, Beijing, China; ^6^Sanqi Research Institute of Yunnan Province, Kunming, China

**Keywords:** *Panax notoginseng*, root rot, rhizosphere soil, root endophytes, metagenomic sequencing, microbial community

## Abstract

*Panax notoginseng* (Burk.) F. H. Chen is a Chinese medicinal plant of the Araliaceae family used for the treatment of cardiovascular and cerebrovascular diseases in Asia. *P. notoginseng* is vulnerable to root rot disease, which reduces the yield of *P. notoginseng*. In this study, we analyzed the rhizosphere soil and root endophyte microbial communities of *P. notoginseng* from different geographical locations using high-throughput sequencing. Our results revealed that the *P. notoginseng* rhizosphere soil microbial community was more diverse than the root endophyte community. *Rhodopseudomonas*, *Actinoplanes*, *Burkholderia*, and *Variovorax paradoxus* can help *P. notoginseng* resist the invasion of root rot disease. *Ilyonectria mors-panacis*, *Pseudomonas fluorescens*, and *Pseudopyrenochaeta lycopersici* are pathogenic bacteria of *P. notoginseng*. The upregulation of amino acid transport and metabolism in the soil would help to resist pathogens and improve the resistance of *P. notoginseng.* The ABC transporter and gene modulating resistance genes can improve the disease resistance of *P. notoginseng*, and the increase in the number of GTs (glycosyltransferases) and GHs (glycoside hydrolases) families may be a molecular manifestation of *P. notoginseng* root rot. In addition, the complete genomes of two *Flavobacteriaceae* species and one *Bacteroides* species were obtained. This study demonstrated the microbial and functional diversity in the rhizosphere and root microbial community of *P. notoginseng* and provided useful information for a better understanding of the microbial community in *P. notoginseng* root rot. Our results provide insights into the molecular mechanism underlying *P. notoginseng* root rot and other plant rhizosphere microbial communities.

## Introduction

*Panax notoginseng* (Burk.) F. H. Chen, belonging to the Araliaceae family, is a Chinese medicinal plant that is mainly grown in Yunnan and Guangxi Provinces of China and is commonly used for the treatment of coronary heart and cardiovascular diseases (Hong et al., 2010; Fan et al., 2016). *P. notoginseng* is often used in a large variety of traditional Chinese medicine. *P notoginseng* has been widely used in China and other Asian countries as a therapeutic strategy or health care product, such as hemostatic, anti-thrombotic, and anti-atherosclerotic agent, as well as used to reduce blood pressure and relieve pain, and presents neuroprotection effects (Yang et al., 2019; Guo et al., 2020). Same as the root of the medicinal part of ginseng, the root of *P. notoginseng* has been used as a raw medicinal material in more than 400 products in 1,300 companies in China (Miao et al., 2006). The World Health Organization has reported that approximately one-third of all deaths worldwide are attributed to cardiovascular diseases (Santhakumar et al., 2018). As the incidence of cardiovascular diseases has increased, and with the modernization of Chinese medicine, the demand for root material of *P. notoginseng* has been increasing. However, *P. notoginseng* is vulnerable to various plant diseases, of which root rot is the most serious, limiting *P. notoginseng* production.

Approximately 70% of medicinal plants have different degrees of pathogen infection related to continuous cropping obstacles (Wu et al., 2013; Zhao Y. P. et al., 2016). The death rates of *P. notoginseng* seedlings were 2.0–39.4 and 2.6–81.2% in continuous cropping and replanting systems, respectively (Dong et al., 2016). Root rot can reduce *P. notoginseng* yield by 70% during the entire growing period (Dong et al., 1988). Previous studies showed that *Cylindrocarpon destructans* var. *destructans* causes root rot in *P. notoginseng* (Mao et al., 2014); *Fusarium oxysporum* and *Fusarium solani* are associated with root rot disease in *P. notoginseng* (Ma et al., 2019). Using the internal transcribed spacer (ITS) sequencing technology, the dominant microorganisms in root rot of *P. notoginseng* was identified as *Ilyonectria mors-panacis* (Mi et al., 2017), and found several potential pathogenic fungi in the rhizosphere soil of 2-year-old healthy *P. notoginseng* plants (Miao et al., 2016). Owing to the influence of various plant diseases, continuous cropping obstacles, and the Daodi (the distinctively higher quality of the medicinal materials that grow in a certain area) of *P. notoginseng* (Liu et al., 2020), the available planting area of *P. notoginseng* is decreasing. Therefore, to increase the production of *P. notoginseng*, it is necessary to identify the key pathogens associated with root rot in this species to provide support for research on corresponding prevention methods.

The microbial community plays important roles in nutrient absorption and resistance to environmental pressures, and the rhizosphere microbial community is also essential for plant health and disease suppression in rhizosphere soil. Previous studies have shown that the rhizosphere soil microbial diversity of healthy plants is greater than diseased plants (Brussaard et al., 2007; Bakker et al., 2010). Recently, it has been shown that plant stress responses are linked to the composition of beneficial plant microbial communities (Bakker et al., 2018). The relationship between soybeans and rhizosphere microbial communities shows that resistant soybeans are rich in genes encoding phenazines, phospholipids, and other antifungal-related genes. Furthermore, more rhizosphere bacteria resistant to the *Fusarium* genotype can be synthesized (Mendes et al., 2018). The microbial communities in the rhizosphere soil and roots of healthy and diseased *P. notoginseng* have been analyzed through bacterial 16S rRNA and fungal 18S rRNA genes. In the rhizosphere soil of diseased *P. notoginseng*, microbial communities showed a decrease in alpha diversity, the bacterial community dissimilarity increased, and fungal community dissimilarity decreased. However, the bacterial and fungal community dissimilarity in the roots were not markedly different between healthy and diseased *P. notoginseng* (Wu et al., 2015). It has also been shown that following root rot infection of *P. notoginseng*, the content of phenolic acid in the soil is lower in diseased *P. notoginseng*, and increase the phenolic acid content can effectively prevent the root rot of *P. notoginseng* (Zhang et al., 2018). Phenolic acid can inhibit the growth of pathogenic bacteria, while also stimulating the production of ferric acid; therefore, adjusting the content of phenolic acid can effectively prevent root rot disease occurrence in *P. notoginseng* (Zhao et al., 2018).

Metagenomics refers to the total DNA of the microbial community investigated at one point, allowing the identification of all microorganisms in an environment, especially those that are difficult to cultivate in a laboratory (Chen and Pachter, 2005). Metagenomics technology is an effective approach to find unculturable microorganisms and overcome the limitation of amplicons (Wang P. P. et al., 2020). Several studies have applied metagenomics technology to help solve disease-related problems in plants, such as potato (Goss et al., 2014), peas (Gaulin et al., 2007; Taheri et al., 2017), citrus (Duan et al., 2009), and tomatoes (Kwak et al., 2018). Previous studies have investigated root rot in *P. notoginseng* using the amplicon technologies, such as 16S/18S rRNA sequencing and ITS, and mostly focused on fungi. Even though other species such as *Panax ginseng* and *Panax quinquefolius* have been studied (Xing et al., 2010; Vendan et al., 2012; Ji et al., 2018; Deng et al., 2020; Wang Q. et al., 2020), reports on the endophytes and rhizosphere microorganisms of *P. notoginseng* remain limited and no studies have investigated the microbial community of root rot *P. notoginseng* using metagenomics.

In this study, the high-throughput sequencing technology was used to sequence the rhizosphere soil and root endophytes of healthy and root rot diseased *P. notoginseng*. We aimed to reveal the structure and functional difference of microbial community through metagenomic analysis and report the pathogenic and antagonistic bacteria of root rot diseased *P. notoginseng*. Investigating the correlation between the root rot and microbial community will provide a theoretical basis for the prevention and treatment of root rot in *P. notoginseng*. Consequently, the findings of this study will ensure the sustainable development of *P. notoginseng*.

## Materials and Methods

### Sample Collection and DNA Isolation

**FIGURE 1:**
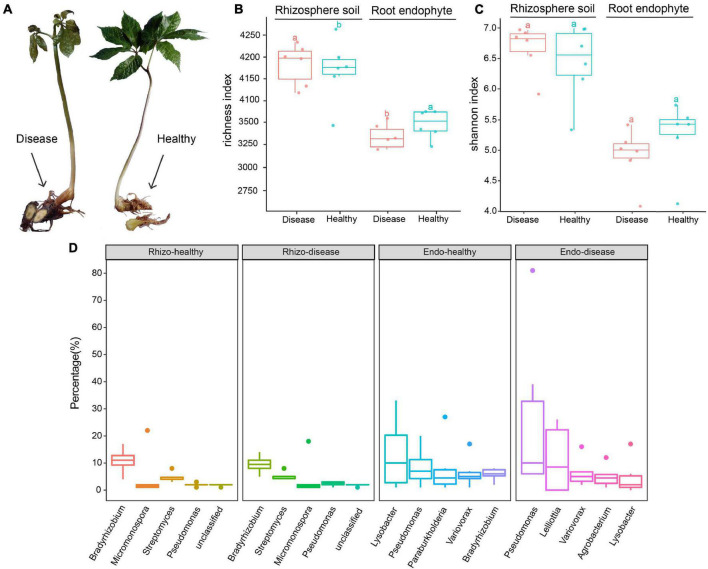
Characteristics of healthy and root rot diseased *Panax notoginseng* plants. **(A)** Diseased and healthy *P. notoginseng* plant parts. **(B)** Observed OTUs box plot based on the rhizosphere soil and root endophyte bacteria species abundance. **(C)** Shannon index box plot based on the rhizosphere soil and root endophyte bacteria species abundance. **(D)** Boxplots of the top five species in each of the rhizosphere soil and root endophyte groups. Tukey’s HSD test is used to compare the differences between two groups and add the difference group letters.

In this study, Lijiang City (LJ) (26°49′59″ N, 100°3′24″ E, altitude: 2,640 m) and Qiubei County (QB) (24°2′49″ N, 103°58′40″ E, altitude: 2,020 m) in Yunnan Province were selected as sampling points of *P. notoginseng*. We set three biological replicates in each sampling point. In each biological replicate, one diseased (the aboveground parts were wilted and yellowed, and the roots were necrotic) and one healthy plant (no signs of root rot) were selected (Figure 1A). In each plant, the root endophyte and corresponding rhizosphere soil were collected. A total of 24 samples were collected for further analysis.

When collecting the root endophyte of *P. notoginseng*, the plant was gently lifted from the soil and shaken to remove the dirt and other impurities attached to the roots. Thereafter, the roots were carefully rinsed with sterile water until the root surfaces were free of impurities. A prepared sterile scalpel was used to cut off the cleaned roots (∼100 g) into 50 mL sterile centrifuge tubes and were immediately placed into liquid nitrogen for preservation. Then, the corresponding rhizosphere soil of the plant was collected. A standard soil ring knife was used to collect the rhizosphere soil (∼20 cm under the ground) from each plant and placed into 50 mL sterile centrifuge tubes that were stored in liquid nitrogen for preservation.

DNA isolation performed using the modified CTAB method (Doyle and Doyle, 1987). Briefly, we piped 1,000 μL CTAB lysate into a 2.0 mL EP tube; added lysozyme and 3 g sample to the lysate; placed the EP tube into a 65°C water bath; inverted and mixed several times to fully mix the sample. Then, we centrifuged to collect the supernatant, added phenol: chloroform: isoamyl alcohol (25:24:1), mixed, and centrifuged at 12,000 rpm for 10 min. Subsequently, we removed the supernatant, added chloroform: isoamyl alcohol (24:1), inverted and mixed, and centrifuged again at 12,000 rpm for 10 min. We placed the supernatant into a 1.5 mL centrifuge tube, added isopropanol, mixed, and left to precipitate at −20°C. Then, we centrifuged at 12,000 rpm for 10 min, removed the liquid and washed the precipitate with 1 mL 75% ethanol. After washing twice, the remaining liquid could be collected after centrifugation and removed with a pipette. We placed the final sample in an ultra-clean workbench to dry at 25°C. We added ddH_2_O to dissolve the DNA sample, 1 μL RNase A was added to digest the RNA, and placed the sample at 37°C for 15 min. The collected DNA sample was used for sequencing.

### Metagenomic Sequencing and Quality Control

A Qubit 2.0 fluorometer (Thermo Fisher Scientific Inc., Waltham, MA, United States) was used to accurately quantify the DNA concentration. After the DNA samples were quantified, they were randomly interrupted using the Covaris Focused-ultrasonicator (Covaris, Inc., Woburn, MA, United States), and then the library was prepared following the steps of end repair, A-tailing, sequencing adapters, purification, and PCR amplification followed by Illumina library construction protocol (Illumina Inc., San Diego, CA, United States). Initially, library was constructed, and the inserted fragments of the library were detected using the Agilent 2100 bioanalyzer (Agilent, Santa Clara, CA, United States). After the size of the inserted fragments were standardized (500 bp), the effective concentration of the library was accurately quantified using the Q-PCR method to ensure the quality of the library. Secondly, libraries were pooled to flow cells according to the effective concentration and target data volume requirements. At last, the cBOT was clustered, a NovaSeq 6000 high-throughput sequencing platform (Illumina, Inc., San Diego, CA, United States) was used for sequencing. Each root endophyte sample had approximately 20–30 Gb of sequencing data, and each rhizosphere soil sample had approximately 10–30 Gb of sequencing data.

The FastQC software was used to estimate the quality of the original sequences of 24 samples to generate a quality evaluation report (Andrews, 2010). The Kneaddata tool^1^ was used to perform quality control and de-hosting the *P. notoginseng* genome sequence. The Kneaddata process relies on Trimmomatic to remove adaptors (Bolger et al., 2014), linkers, and low-quality sequences (parameters: sequence quality ≥ 20, minimum sequence length ≥ 50 bp). Bowtie2 (Langmead and Salzberg, 2012) was used to mapping the reads to the genome of *P. notoginseng* (Zhang et al., 2017), and reserved the unmapped reads as finally clean sequences (Table 1). Cleaned reads were used for downstream analyses.

**TABLE 1:** Summary table of samples and sequencing data.

Sample type	Plant status	Samples number	Raw reads (Gb)	Clean reads (Gb)
Root endophyte	Disease	LJ-1	25.7	10.9
		LJ-2	32.1	24.9
		LJ-3	25.5	4.16
		QB-1	33.3	17.4
		QB-2	30.4	22.8
		QB-3	31.8	28.1
	Healthy	LJ-1	12.8	1.7
		LJ-2	27.9	4.0
		LJ-3	28.6	6.5
		QB-1	28.5	8.6
		QB-2	27.0	13.6
		QB-3	30.3	9.2
Rhizosphere soil	Disease	LJ-1	13.5	12.8
		LJ-2	13.2	12.8
		LJ-3	13.0	12.5
		QB-1	13.0	12.4
		QB-2	12.7	12.3
		QB-3	13.1	12.6
	Healthy	LJ-1	33.9	32.5
		LJ-2	10.5	10.1
		LJ-3	13.0	12.5
		QB-1	12.2	11.7
		QB-2	12.8	12.3
		QB-3	12.9	12.3

*Raw reads are the data volume of paired-end sequencing, and clean reads are sequences that are still paired after quality control and de-hosting.*

### Analysis of the Microbial Diversity of Root Rot *Panax notoginseng*

The HUMAnN2 software was utilized to calculate the species and functional composition of the samples (Franzosa et al., 2018). HUMAnN2 does not consider paired-end information in the analysis, therefore the paired-end sequence after quality control is combined as one input file, while the other parameters are the default parameters. MetaPhlAn2 was used to calculate the species composition (Truong et al., 2015). We mapped the clean reads to the HUMAnN2 nucleic acid database using Bowtie2 software (Langmead and Salzberg, 2012), and generated species abundance composition table for each group. We used GraPhlAn to analyze the top 100 representative species in the species composition table at the phylum level (Asnicar et al., 2015). To display the species circle map, R software version 3.4.3 was used to display the heat map of the species at the genus level (R Core Team, 2015, 2020), and to compare the differences in species composition between groups. Finally, LEfSe was used for the species difference analysis (Segata et al., 2011). The LEfSe used the Kruskal–Wallis and Wilcoxon signed-rank tests, signed linear judgment analysis (LDA) logarithmic score, and related *P*-values to identify species with significant differences in each group (LDA > 4 was considered as significantly different species) (Zuo et al., 2021).

Kraken2 is a high-precision metagenomic sequence classification software based on the *k*-mer algorithm (Wood et al., 2019). Kraken2 is used for species annotation at the read level, which can rapidly classify sequencing reads for species classification. We compared our data to the Kraken2 standard library, fungi, protozoa, and plasmid databases. The bacteria, archaea, fungi, protozoa, plasmids, and virus libraries were provided by the RefSeq database^2^. The species composition table was used for species diversity analysis, the species level annotations were extracted and combined to the minimum sequencing amount, and alpha diversity was calculated using the R. In addition, alpha diversity boxplots and the top 30 genera of each group were used to compare the differences.

The EukDetect method (Lind and Pollard, 2021) was used to detect eukaryotic microorganisms in the clean data. The EukDetect pipeline uses a Snakemake workflow engine (Kster and Rahmann, 2018). Microbial eukaryotic genomes were downloaded from NCBI GenBank for all species designated as fungi, protists, other non-vertebrate metazoans, and non-streptophyta archaeplastida species. In addition to the GenBank genomes, 314 genomes and transcriptomes comprising 282 protists, 30 *Archaeplastida* species, and 2 metazoans curated by the EukProt project were downloaded (Richter et al., 2020).

### Assembly and Binning Analysis of Metagenomic Data

MEGAHIT (Li et al., 2015) was used for each sample assembly. The contigs of each sample were mixed by group, and the quality of contigs was assessed by QUAST (Alexey et al., 2013). MetaProdigal (Hyatt et al., 2012) was used for gene prediction, and cd-hit-est (Fu et al., 2012) to cluster and de-redundancy the predicted genes to construct a non-redundant gene set (similarity ≥ 95%, coverage ≥ 90%). The non-redundant gene set obtained was used for subsequent analysis. The nucleic acid sequences were quantified using Salmon (Patro et al., 2017). The parameter was set to the metagenomic model (*–meta*) for all reads that could be binned, and the abundance of genes in each sample was obtained.

We used MetaWRAP (Uritskiy et al., 2018) to excavate the draft genome of a single strain. This process integrates three popular binning software. We selected the MaxBin2 (Wu et al., 2016) and MetaBAT2 (Kang et al., 2019) software for binning. The de-redundant contigs of each group were binned, and the bins were purified. We evaluated and comprehensively analyzed the results to obtain better results. The Checkm database (Parks et al., 2015) requires completeness of more than 70% and a pollution rate of less than 5%, which was used for purification. After purification, the Blobology module was used to visualize the GC content and abundance of contigs by comparing the NCBI nt and taxonomy database was obtained. Samlon (Patro et al., 2017) was used to quantify the bins and calculate the abundance of a single bacterial genome in each sample. Subsequently, each contig in the bin was annotated, and the species of each bin was estimated. Krona was used to visualize the results of bin annotation (Ondov et al., 2011). Finally, Bins were classified using a concatenated set of 122 universal marker proteins by GTDB-Tk to find new species (Chaumeil et al., 2019).

### Functional Annotation

Diamond (Huson and Buchfink, 2015) was used to compare contigs to the UniProt protein library^3^, which includes the UniRef90 protein database; the MetaCyc metabolic pathway database was used to predict gene pathways^4^. MinPath^5^ defines the minimum set of pathways, producing results at the protein, gene, and pathway levels, and produces a functional composition table. The STAMP (Parks et al., 2014) software was used to calculate the functional pathways and to produce a histogram. Significant functional differences between the groups were set at *P* < 0.05.

The nucleic acid sequence was translated into a protein sequence and compared to the eggNOG (Jaime et al., 2016) database using the Diamond (Huson and Buchfink, 2015). The eggNOG database integrates annotation information, such as Gene Ontology (GO), clusters of orthologous genes (COG), and KEGG orthologs (KO) information. We performed multiple sequence alignment between the query and the eggNOG database to determine the conserved sites and analyze their evolutionary relationships (Jaime et al., 2016). We selected the possible gene names of each sequence predicted by the eggNOG database and eliminated the duplicated results. The eggNOG annotation results were used for subsequent analysis. Diamond (Huson and Buchfink, 2015) was used to align the protein sequence with the carbohydrate-active enzymes database (CAZy) (Vincent et al., 2013). The CAZy database provides an understanding of the nature and extent of the complex carbohydrate metabolism focuses on the differences in carbon source metabolism between species, and was used to compare to the Resfams database using Diamond (Gibson et al., 2015), we predicted the unknown resistance gene and obtained the mechanism and function of the resistance genes in each sample.

### *In vitro* Validation of *Pseudomonas fluorescens*

The *Pseudomonas fluorescens* strain (Mingzhou Biotechnology Co., Ltd., Ningbo, China) was expanded using NA (Nutrient Agar) culture medium. Before the experiment, the healthy *P. notoginseng* was scratched with a sterile knife to facilitate the entry of *P. fluorescens* into the plant. A control and an experimental group were set up in this study. The diluted *P. fluorescens* strain was added into the culture bottle with healthy *P. notoginseng* in the experimental group. The control group was filled with sterile water with the same volume, and each group had three biological replicates, all samples were cultured at 25°C, and the results were observed each day.

## Results

### Metagenomic Sequencing Data Quality Control and Saturation Verification

A total of 333.90 Gb raw data were produced from the 12 root endophyte samples, with an average of 27.83 Gb raw data per sample. Twelve rhizosphere soil samples produced a total of 173.80 Gb raw data with an average of 14.48 Gb raw data per sample. After assessing the quality of the original data, the Q30 values of all samples were >93%. Quality controls were performed on the raw data to remove host contamination and obtain clean data. After removing the host-related sequences of *P. notoginseng*, each rhizosphere soil and root endophyte sample had an average of 13.90 and 12.66 Gb of clean data (Table 1 and Supplementary Table 1). Based on the alpha diversity chao1 and observed dilution curves (Supplementary Figure 1) of rhizosphere soil and root endophyte samples, Supplementary Figures 1A,B show that the data were saturated at 500,000 sequences (0.075 Gb) of rhizosphere soil sample. Similarly, Supplementary Figures 1C,D show saturation at 1,000,000 sequences (0.15 Gb) of the root endophyte sample. These results confirm that the sequencing depth of each sample reached saturation, enabling us to perform the next steps of this study.

### Microbial Community Characteristics Between Healthy and Diseased *Panax notoginseng*

Kraken2 is a high-precision metagenomic sequence classification software based on the *k*-mer algorithm, which can classify sequencing data by species (Wood et al., 2019). In this study, the species diversity of each group was analyzed, the alpha diversity and Shannon index boxes were plotted (Figures 1B,C). The other diversity index boxes are shown in Supplementary Figure 2. Boxplots of the top five genera are shown in Figure 1D. The results showed that the overall richness of the rhizosphere soil was higher than the root endophytes. The number of operational taxonomic units (OTUs) in the rhizosphere soil of diseased plants was higher than healthy plants, whereas, in the root endophyte samples, the number of OTUs of healthy plants was higher than diseased plants (Figure 1B). The abundance of *Bradyrhizobium* in healthy rhizosphere soil was higher than in diseased rhizosphere soil (Figure 1D). In the healthy rhizosphere soil, the top three genera were *Bradyrhizobium*, *Micromonospora*, and *Streptomyces*. The genus-level differences were more evident in the root endophytes between healthy and diseased plants. The three most abundant genus in the healthy root endophytes were *Lysobacter*, *Pseudomonas*, and *Paraburkholderia*. The top three genera in diseased root endophytes were *Pseudomonas*, *Lelliottia*, and *Variovorax*. *Lysobacter* and *Paraburkholderia* were more abundant in healthy samples than in root rot diseased samples, whereas *Pseudomonas* and *Lelliottia* were more abundant in diseased root endophyte samples (Figure 1D).

**FIGURE 2:**
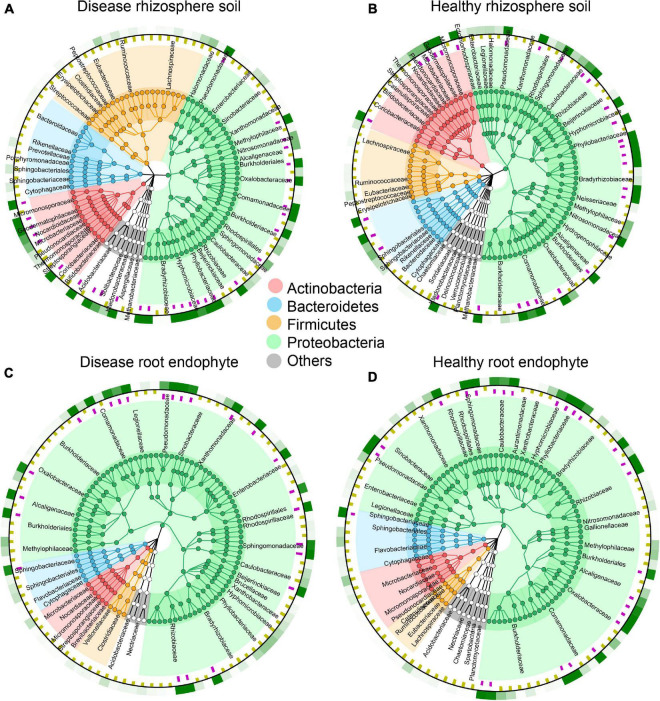
Species composition circle diagrams based on the top 100 most abundant species in rhizosphere soil and root endophyte samples. The colors represent the four phyla that contain these species. The purple squares in the first ring indicate the species with an abundance greater than 5 per 1,000. The yellow squares in the second ring indicate the species with an abundance less than 5 per 1,000. The third ring indicates the average value for all samples, where the different shades of green show the different species abundances. **(A)** Top 100 most abundant species in disease rhizosphere soil. **(B)** Top 100 most abundant species in healthy rhizosphere soil. **(C)** Top 100 most abundant species in disease root endophyte. **(D)** Top 100 most abundant species in healthy root endophyte.

**FIGURE 3:**
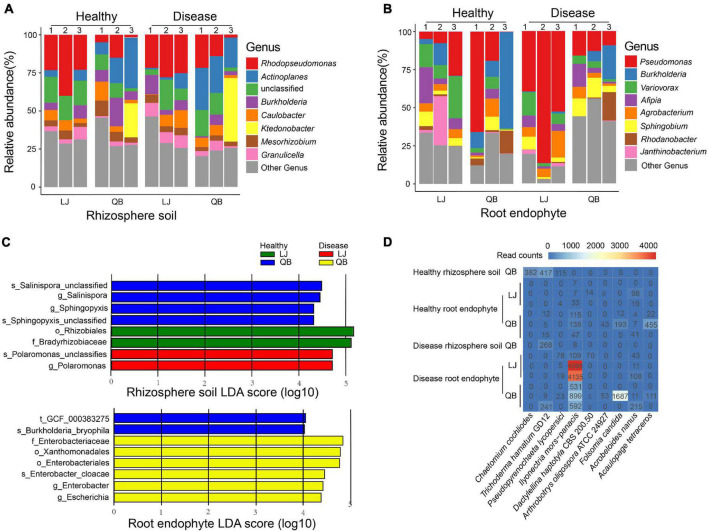
Genus composition heatmaps and corresponding histograms for the microbial communities associated with healthy and root rot *Panax notoginseng*. **(A,B)** Relative abundance of the top eight species in each sample. **(C)** Species differences in the rhizosphere soil and root endophyte microbial communities associated with healthy and root rot *P. notoginseng*. Display the species with significant differences (LDA > 4) between the groups. Panel **(D)** shows the top nine species of eukaryotes present in the samples. Lijiang (LJ) and Qiubei (QB) and 1, 2, and 3 represent biological replicate in each location.

We used the species annotation results of MetaPhlAn2, which is more accurate. There are 143 OTUs in rhizosphere soil and 187 OTUs in root endophyte, and we chose the top 100 species, which is more representative and classified them by phylum. As indicated by the colors in the species circle diagrams, there were four main phyla: *Actinobacteria*, *Bacteroides*, *Firmicutes*, and *Proteobacteria*. Species that did not classify into these four phyla were grouped as others in the circle diagrams. The circle diagrams showed that *Proteobacteria* occurred in the highest proportion in both the rhizosphere soil and root endophyte samples. The relative occurrence of the phyla in the rhizosphere soil and root endophyte samples are *Actinobacteria*, 14 and 7%; *Bacteroides*, 10 and 7%; *Firmicutes*, 20 and 6%; *Proteobacteria*, 50 and 77%; others, is 6 and 3%, respectively (Figure 2). The species composition histograms showed the differences at the genus level between different health statuses (Figure 3). A total of 87 genera were detected in the rhizosphere soil and the top seven most abundant genera were *Rhodopseudomonas*, *Actinoplanes*, *Burkholderia*, *Caulobacter*, *Ktedonobacter*, *Mesorhizobium*, and *Granulicella* (Figure 3A). The rhizosphere soil bacterial community in the diseased and healthy rhizosphere soil samples from LJ and QB showed contrasting results. The abundance of *Rhodopseudomonas* was higher in the healthy rhizosphere soil at the LJ site, whereas it was more abundant in the root rot diseased rhizosphere soil at the QB site. *Ktedonobacter* was found only in the rhizosphere soil from QB and was more abundant in the root rot diseased soil sample. In total, 108 genera were detected in the root endophyte samples (Figure 3B). The eight most abundant genera were *Pseudomonas*, *Burkholderia*, *Variovorax*, *Afipia*, *Agrobacterium*, *Sphingobium*, *Rhodanobacter*, and *Janthinobacterium*. *Pseudomonas* showed a high abundance in the diseased root endophyte samples from LJ, and a high abundance in the healthy root endophyte samples from QB, which may be related to the ecological environment of the different locations. The abundance of *Burkholderia* in the healthy root samples was higher than in diseased samples. *Variovorax* and *Afipia* showed high abundance in the healthy root endophyte samples from LJ. Overall, the abundance of *Agrobacterium* and *Sphingobium* in the diseased root endophyte samples was higher than in healthy samples. There were eight species with significant differences in the root endophyte and rhizosphere soil samples (LDA > 4 for significant differences; Figure 3C).

We also analyzed the composition of fungi in all samples and a total of 12 eukaryote species in the rhizosphere soil, and 29 in the root endophyte samples were present (Supplementary Table 2). *Acaulopage tetraceros*, *Acrobeloides nanus*, *Arthrobotrys oligospora* ATCC 24927, *Dactylellina haptotyla* CBS 200.50, and *Folsomia candida* were unique to root endophytes; *A. tetraceros* and *Chaetomium cochliodes* were more abundant in the healthy samples; *A. nanus*, *A. oligospora* ATCC 24927, *F. candida*, and *Pseudopyrenochaeta lycopersici* were more abundant in the diseased samples; *I. mors-panacis* was the most abundant species in the diseased samples (Figure 3D).

### Functional Composition and Pathways of Healthy and Root Rot *Panax notoginseng*

**FIGURE 4:**
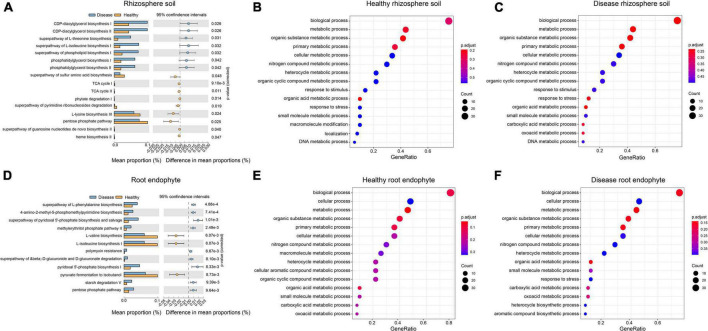
Differences in the functional composition of the rhizosphere soil and root endophyte microbial communities associated with healthy and root rot *P. notoginseng*. Panels **(A,D)** show the functional pathways with significant differences between healthy and diseased samples, respectively. Panels **(B,C,E,F)** represent the Gene Ontology (GO) enrichment annotation results for each group.

There were 16 significantly different pathways in the rhizosphere soil. The pathways of the CDP-diacylglycerol biosynthesis I/II, superpathway of L-threonine biosynthesis, and superpathway of L-isoleucine biosynthesis I were significantly up-regulated in the diseased rhizosphere soil samples, whereas the L-lysine biosynthesis III and pentose phosphate pathway were significantly up-regulated in the healthy rhizosphere soil (Figure 4A). There were 12 significantly different functional pathways in the root endophyte samples. Superpathway of L-phenylalanine biosynthesis, 4-amino-2-methyl-5-phosphomethylpyrimidine biosynthesis and pyridoxal 5′-phosphate biosynthesis I was significantly up-regulated in the root endophyte diseased sample, whereas in the healthy root endophyte samples L-valine biosynthesis, L-isoleucine biosynthesis I, and pyruvate fermentation to isobutanol were significantly up-regulated (Figure 4D). The GO term annotation results were mainly divided into three categories: molecular function (MF), cellular component (CC), and biological process (BP). The top 50 GO terms of each group were classified and enriched, and the identified GO terms belonged to the BP category (Figure 4). The enrichment degree of the GO terms in the root endophytes (Figures 4E,F) was higher than that in the rhizosphere soil (Figures 4B,C). The pathways of the top three enrichments in the rhizosphere soil were the BP, metabolic process, and organic substance metabolic process. The carboxylic acid metabolic process and the oxoacid metabolic process were enriched in the root rot diseased soil group and did not appear in the top 20 processes associated with the healthy rhizosphere soil (Figures 4B,C). The macromolecule metabolic process, cellular aromatic compound metabolic process, and organic cyclic compound metabolic process were significantly enriched in healthy root endophytes and did not occur in the top 20 processes of the diseased root endophytes (Figures 4E,F).

We *de novo* assembled the clean data, evaluated the quality of the contig, and then performed de-redundancy, quantification and obtained pure contig for subsequent functional annotation. We obtained 11.22 Gb contigs with an average contig N50 of 1, 287 bp (Supplementary Table 3). We compared the contigs to the eggNOG (Jaime et al., 2016) database, integrated the annotation results, and counted the annotated COG orthologous proteins in each group. The COG classified 24 functional descriptions, and the annotation implied no significant differences among the 24 functional descriptions of the diseased and healthy rhizosphere samples (*P* > 0.05). The three dominant functions with higher abundance in the rhizosphere soil samples were S: function unknown (healthy rhizosphere soil = 20.61%, diseased rhizosphere soil = 20.85%), E: amino acid transport and metabolism (healthy rhizosphere soil = 9.82%, diseased rhizosphere soil = 9.54%), and C: energy production and conversion (healthy rhizosphere soil = 8.69%, diseased rhizosphere soil = 8.22%; Supplementary Table 3). Among the 24 functional descriptions of the root endophytes, four of them were significantly different between the diseased and healthy samples (*P* < 0.05), and all of them showed a higher abundance in the diseased samples: E: amino acid transport and metabolism (*P* = 0.011), N: cell motility (*P* = 0.028), O: posttranslational modification, protein turnover, chaperones (*P* = 0.027), and S: function unknown (*P* = 0.027) (Supplementary Figure 3A). The three dominant functions with a higher abundance in the root endophyte samples were S: function unknown (healthy root endophyte = 20.73%, diseased root endophyte = 19.38%), E: amino acid transport and metabolism (healthy root endophyte = 8.88%, diseased root endophyte = 9.60%), and P: inorganic ion transport and metabolism (healthy root endophyte = 8.29%, diseased root endophyte = 8.63%), the functional pathway of which was relatively abundant in the root rot diseased samples (Supplementary Table 3).

The Resfam database analysis results indicated that there were 15 classifications of mechanisms with 94 functional descriptions. Among them, the diseased rhizosphere soil group had the most resistance genes (106,554), with an average of 17,759 resistance genes per sample. The resistance genes of the other groups were healthy rhizosphere soil (89,250), diseased root endophyte (84,135), and healthy root endophyte (57,122). Overall, there were more resistance genes in the rhizosphere soil than in the root endophytes, and the number of resistance genes in the diseased group was higher than in the healthy group. The ABC transporter, RND antibiotic efflux and gene modulating resistance functions in the root rot disease group were higher than those in the healthy group (Supplementary Table 3 and Supplementary Figure 3B).

The KO function results for each group were counted. Among them, the healthy rhizosphere soil group had a total of 7,898 KOs, the disease rhizosphere soil group presented 8,078 KOs, the healthy root endophyte group presented 9,886 KOs, and the disease root endophyte group presented 10,736 KOs. The number of KOs in the root endophytes was higher than in the rhizosphere soil, and that of KOs in the diseased group was higher than in the healthy group. There was 6,469 core KOs in the four groups, accounting for 54.4% (Supplementary Figure 3C). In addition, each group had unique KOs, which were 106 (0.9%), 124 (1.0%), 435 (3.7%), 921 (7.7%), sorted by healthy rhizosphere soil, disease rhizosphere soil, healthy root endophyte, and disease root endophyte, respectively. The KO results showed that zearalenone synthase (K15418), lysophospholipid acyltransferase (K13519), Pin2-interacting protein (K11135), G protein-coupled receptor 64 (K08451), and MFS transporter (K08181) are unique KO in disease root endophyte.

The CAZy database includes five categories and one related module. Each category was divided into families. Among them, the glycoside hydrolases (GHs), which mainly hydrolyze and rearrange glycosidic bonds, presented a total number of 167 families (Vincent et al., 2013). We found 123 GH families in the healthy rhizosphere soil, 127 in the diseased rhizosphere soil, 126 in the healthy root endophyte, and 131 in the diseased root endophyte group (Supplementary Table 3). The number of GH families in the diseased samples was slightly higher than that in the healthy group. Similarly, there were 110 families of glycosyltransferases (GTs), mainly involved in the formation of glycosidic bonds. In addition, polysaccharide lyases (PLs) presented 40 families, carbohydrate esterases have 17 families, auxiliary activities have 16 families, and carbohydrate-binding modules have 86 families. These families were observed in slightly higher numbers in the diseased group than in the healthy group. The sequence annotation results of CAZy database showed that healthy rhizosphere soil, disease rhizosphere soil, healthy root endophyte, and disease root endophyte show an increasing trend by turns. The healthy rhizosphere soil group had the lowest number of reads matched to the CAZy database, the diseased root endophyte group had the most reads matched to the CAZy database, and the GT family matched ratio was the highest, followed by that of the GH family (Supplementary Table 3).

### Metagenomic Data Binning

After assembling the clean data, de-redundant contigs were binned to mine the single bacterial genome. Ultimately, 9 bins were obtained in the healthy rhizosphere soil group, 24 bins in the diseased rhizosphere soil group, and 2 bins in the diseased rhizosphere soil group with >99% completeness. *Thaumarchaeota* had 99.51% completeness with the genome length of 1.40 Mb; *Clostridium* had 99.01% completeness with the genome length of 6.31 Mb in the diseased rhizosphere soil (Supplementary Figure 4 and Supplementary Table 4). A total of 41 bins were in the healthy root endophyte group, two of which were 100% complete, namely *Flavobacteriaceae* (genome length = 5.00 Mb) and *Bacteroidetes* (genome length = 6.34 Mb). There were 91 bins in the diseased root endophyte group, one of which was 100% complete (*Flavobacteriaceae*, genome length = 4.3 Mb). We found that these two 100% complete *Flavobacteriaceae* genomes were two different species under the *Flavobacteriaceae* genus through similarity comparison. The GC content results indicated that the abundance of orders in the rhizosphere soil was greater than that in the root endophytes (Supplementary Table 4). These results are consistent with the findings of higher microbial diversity in the rhizosphere soil than root endophytes samples (Figure 1B).

The abundance of the purified single bacterial genome in each sample was determined using bin quantification, and the results are displayed in a heat map (Supplementary Figure 4). Bacteria (bin 2) were enriched in the QB-3 sample and *Sphingomonadaceae* (bin 13) was enriched in the QB-1 sample (Supplementary Figures 4A,C). *Alphaproteobacteria* (bin 3) were enriched in the LJ diseased root endophyte samples (Supplementary Figure 4D and Supplementary Table 4). Based on taxonomic annotations from the Genome Taxonomy Database (GTDB), the analysis revealed that the bins cover 34 known species (ANI > 95%). There are 132 bins with <95% ANI of the existing genome, which represented potential new species. The GTDB classification of all the 166 bins is shown in Supplementary Table 4.

## Discussion

### Microbial Communities in Different Ecological Niches of Root Rot *Panax notoginseng*

The shape of the dilution curves for the rhizosphere soil and root endophyte samples significantly differed (Supplementary Figure 1). Among the rhizosphere soil samples (Supplementary Figures 1A,B), only the healthy rhizosphere soil dilution curve shape changed, while the dilution curves of the diseased rhizosphere soil samples remained uniform and the shape of the root endophyte dilution curves was more distinct (Supplementary Figures 1C,D). The evident change in the OTU dilution curve of root endophytes may be attributed to the uneven distribution of the microbial community associated with the roots and other plant parts (Gottel et al., 2011). In addition, when comparing the observed OTUs of rhizosphere soil and root endophyte samples (Figure 1B), we found that the microbial diversity of the rhizosphere soil was higher than that of the root endophytes. Studies have shown that the microbial community of rhizosphere soil is more diverse than root endophytes (Johnson and Jumpponen, 2005; Bulgarelli et al., 2013), with a clear compartmental separation between the rhizosphere soil and root endophyte of plants (Bulgarelli et al., 2012, 2015; Lundberg et al., 2012; Peiffer et al., 2013; Zarraonaindia et al., 2015; Yeoh et al., 2016; Hartman et al., 2017). Our results illustrate this uniqueness of the microbial communities (Figures 3A,B). Previous studies have also shown that the bacteria of root endophytes are recruited from the rhizosphere soil (Chaparro et al., 2014; Edwards et al., 2015), which may explain the higher microbial diversity in the rhizosphere soil than root endophyte.

### Bacterial Community Differences Between Healthy and Root Rot *Panax notoginseng*

We compared the bacterial community difference in rhizosphere soil between healthy and diseased samples and found that *Rhodopseudomonas* was the most abundant in the rhizosphere soil (Figure 3A), especially in the healthy samples from the LJ site. *Rhodopseudomonas* can promote plant growth, and induce resistance in plants (Hsu et al., 2021). In tobacco, *Rhodopseudomonas* induces resistance against tobacco mosaic disease (Su et al., 2017). KTSSR54, an active compound produced by *Rhodopseudomonas palustris*, can help rice inhibit pathogens (Nookongbut et al., 2020). Our results are in line with previous findings showing that *Rhodopseudomonas* can protect *P. notoginseng* from root rot. Among the bacteria studied, *Actinoplanes* have attracted increasing attention since they can inhibit plant pathogens and produce more than 100,000 compounds, such as antifungal compounds, siderophores, hydrocyanic acid, hydrolytic enzymes, and ammonia. *Actinoplanes* can accelerate the absorption of nutrients and have positively influenced plant growth (Palaniyandi et al., 2013; Sreevidya et al., 2016; Sumaira et al., 2016). *Actinoplanes* produce many antibiotics, accounting for approximately 45% of the antibiotics on the market (Liu et al., 2012), most of which can treat plant disease (Samac et al., 2003; Cheng et al., 2010; Zucchi et al., 2010; Palaniyandi et al., 2011). In our study, *Actinoplanes* were more abundant in LJ and QB rhizosphere soil samples (Figure 3A), suggesting that the diseased *P. notoginseng* plants may have recruited *Actinoplanes* to resist root rot pathogens, thereby increasing the abundance of *Actinoplanes* in the soil of diseased plants. *Burkholderia* was relatively abundant in most samples. Several studies have shown that *Burkholderia* has antibacterial properties, promotes plant growth and is a potential and effective biological control agent (Jeong et al., 2003; Kunakom and Eustáquio, 2019). *Burkholderia* is an antagonist of black spot disease in cherries (Ding et al., 2021), and secretes beneficial compounds to protect bananas against *Fusarium wilt* (Xu et al., 2020). In our study, the abundance of *Burkholderia* in the healthy root endophyte samples was higher than that in the diseased samples, which is consistent with previous studies (Jeong et al., 2003; Kunakom and Eustáquio, 2019; Xu et al., 2020; Ding et al., 2021), suggesting that *Burkholderia* is a beneficial bacterium for *P. notoginseng*. We infer that *Burkholderia* can protect *P. notoginseng* from plant diseases.

*Pseudomonas*, the most abundant dominant bacterial species in the root endophyte samples in our study, was more abundant in the diseased samples from LJ than in other groups (Figure 3B). *Pseudomonas* has been previously suggested to cause root rot (Miao et al., 2006), and was only identified at the genus level. Herein, we identified *Pseudomonas* to the species level, with *P. fluorescens*, known to protect many crop plants against soil-borne diseases caused by phytopathogens (Silby et al., 2009; Liang F. et al., 2020), at the highest abundance. It has also been shown that *P. fluorescens*, as a biological control agent, can effectively reduce root knot nematode infestation of cucumbers and replace chemical measures for nematode control, thereby increasing cucumber yield and reducing cultivation costs (Panpatte et al., 2020). Screened under *in vitro* conditions, *P. fluorescens* has been shown to have antagonistic activity against pea root rot, with high biological control activity (Godebo et al., 2020). A recent report found that *Pseudomonas* is recruited from roots to resist plant pathogens and was used to verify the disease incidence of plants where it reduces the disease incidence of *Arabidopsis* (Wen et al., 2020). However, the role of *P. fluorescens* is still controversial. In this study, we conducted an *in vitro* experiment to verify whether *P. fluorescens* has a pathogenic effect on *P. notoginseng* (Supplementary Figure 5). Combining our data with experimental results, we concluded that *P. fluorescens* has a pathogenic effect on *P. notoginseng*. Our results revealed that *Variovorax* had a higher abundance in most healthy root endophytic samples. We identified *Variovorax* to the species level and found that the species with the highest abundance was *Variovorax paradoxus*. *Variovorax* can facilitate the interaction between plants and microorganisms by manipulating plant hormone levels to balance the growth of plant roots. *V. paradoxus* can significantly inhibit potato tuber soft rot and is considered to be a new type of potato tuber soft rot biological control agent (Ha et al., 2018). In our study, the abundance of *V. paradoxus* in healthy root endophyte samples was high. This suggests that *V. paradoxus* may promote the growth of *P. notoginseng* and may be an effective biological control agent for root rot disease.

### Eukaryotic Microbial Communities Associated With Healthy and Root Rot *Panax notoginseng*

Our analysis showed that *I. mors-panacis* had the highest ratio in the diseased root endophyte samples (Figure 3D) corroborating with the findings of a previous study that presented *I. mors-panacis* as the main pathogen of *P. notoginseng* root rot (Mi et al., 2017). *I. mors-panacis* is also the main pathogen of *P. ginseng* root rot (Desrochers et al., 2020; Farh et al., 2020). *P. lycopersici* is also a plant fungal pathogen that causes severe root rot and root knot disease (Errico et al., 2019). In our study, *P. lycopersici* had a higher number of reads in the diseased samples, except for disease rhizosphere soil from QB. *P. lycopersici* has not previously been reported in association with root rot *P. notoginseng*, hence we infer that *P. lycopersici* may be a pathogen of *P. notoginseng* root rot. *A. oligospora* ATCC 24927, a fungus that feeds on nematodes, is often used as a biological control agent for plant and animal parasitic nematodes (Ahman et al., 2002). The number of reads of this fungus was relatively high in the diseased samples in our study. *A. nanus* is a type of nematode that feeds on bacteria and was mainly associated with diseased samples in our study. This could account for the high occurrence of *A. oligospora* ATCC 24927, which feeds on nematodes, in the diseased samples.

### The Interaction of Functional Genes and Pathways Helps Improve Plant Resistance

Amino acids play an important role in plant growth as well as pathogen infection as they are indispensable nitrogen sources for many nutritional pathogens (Douglas, 1993; Rico and Preston, 2008), hence regulating the content of amino acids is critical for the growth and defense of plants. It has been demonstrated that the increased expression of usually multiple acids moving in and out transporters (UMAMIT) can induce plant resistance to pathogens (Besnard et al., 2021). In addition, the existence of the amino acid-sensing mechanism in plants indicates that changing the level of amino acids by changing the metabolism or transport of amino acids may trigger a defense response; furthermore, resistance-related genes are up-regulated (Yang et al., 2014). Our study found higher amino acid transport and metabolism in healthy rhizosphere soil than in diseased rhizosphere soil. Based on previous findings, we concluded that the upregulation of amino acid transport and metabolism in soil and the enhancement of transport activity would help to resist the invasion of pathogens and improve the resistance of *P. notoginseng* (Supplementary Table 3). In a study on the resistance signal mechanism of the related tomato root knot nematode disease, the protein with the greatest difference in the differentially expressed protein group belonged to energy production and conversion because the defense response is an energy-consuming process, which may play a key role in the plant’s disease resistance process (Zhao W. et al., 2016). In our study, the energy production and conversion process in diseased roots was three times that of healthy roots and may be closely related to the disease resistance process of *P. notoginseng* (Supplementary Table 3).

### The Antibiotic Resistance-Related Gene Can Help *Panax notoginseng* Resist Root Rot

The misuse of antibiotics has contributed to the widespread development of antimicrobial resistance among clinically significant bacterial species, therefore, understanding the mechanism of antibiotics in *P. notoginseng* is important for facilitating the healthy growth of this plant species. A previous study has shown that Chinese herbal medicines, such as *P. notoginseng*, can produce compounds that interfere with quorum sensing, which can effectively inhibit pathogens and help delay antibiotic resistance (Koh and Tham, 2011). In our study, ABC transporter (34.16%), gene modulating resistance (20.55%), and RND antibiotic efflux (16.62%) accounted for the highest proportions and the highest number of diseased samples (Supplementary Figure 3B). ABC transporter reactions play major roles in disease resistance of plants (Devanna et al., 2021). The G and B subfamily of the ABC transporter family have been the most researched on related secondary metabolism and is also the key transport family to the defensive process (Hwang et al., 2016; Borghi et al., 2019; Dhara and Raichaudhuri, 2021). The proportion of ABC transporters in the two groups of diseased samples was higher than that in the corresponding two groups of healthy samples, which may be associated with the disease resistance of *P. notoginseng*. Previous studies have reported that gene modulating resistance helps plants to inhibit pathogenic bacteria and improve plant resistance (Gong et al., 2020; Liang Y. et al., 2020; Wang J. et al., 2020; Corredor-Moreno et al., 2021; Zia et al., 2021), while the RND antibiotic efflux mechanism has an efflux effect on various antibiotics; inhibiting these pumps can mitigate antibiotic resistance (Siriyong et al., 2017; Liang et al., 2019; Ichinose et al., 2020). Our study showed that the RND antibiotic efflux mechanism in diseased samples was greater than that in healthy samples. We speculate that the RND antibiotic efflux mechanism in the diseased samples was active and had an efflux effect on antibiotics. Thus, the reduction of antibiotics in the diseased samples might have weakened the resistance to pathogens. This phenomenon may induce plant roots to produce antibiotic efflux, making it easier for root rot to invade.

### *Panax notoginseng* Root Rot Carbohydrate-Active Enzymes Genes

According to the CAZy database comparison results, the proportion of GHs in each group was the largest, and the proportion in diseased samples was higher than that of healthy samples. In a study of peanut stem rot caused by fungi, the number of GHs was the highest in the pathogenic secreted protein (Iquebal et al., 2017). In addition, in soybean seed rot, 149 plant cell wall-degrading enzymes were detected, most of which were GHs (Li et al., 2018). Our results are in line with these previous reports. The number of GHs in the diseased samples was higher than healthy samples, suggesting that the root rot-affected parts of *P. notoginseng* secrete more enzymes related to the GH family. Regarding the GTs, in a study of wheat infection with *F. wilt*, the GTs in wheat infected with *Fusarium graminearum* showed an increasing trend (Ha et al., 2016), indicating that infection with *P. notoginseng* root rot can cause GTs to increase. In short, the increase in the number of GH and GT families can predict the tendency of *P. notoginseng* to suffer from root rot (Supplementary Table 3).

## Data Availability Statement

The original contributions presented in the study are publicly available. This data can be found here: National Center for Biotechnology Information (NCBI) BioProject database under accession number PRJNA753694.

## Author Contributions

PW and YL wrote and reviewed the manuscript and draw the picture. PW, LY, JS, and YL collected the samples. YY, YQ, CW, DL, and LH provided the analysis ideas and reviewed the manuscript. XC and YL supervised the work and read and approved the final manuscript. All authors contributed to the article and approved the submitted version.

## Conflict of Interest

The authors declare that the research was conducted in the absence of any commercial or financial relationships that could be construed as a potential conflict of interest.

## Publisher’s Note

All claims expressed in this article are solely those of the authors and do not necessarily represent those of their affiliated organizations, or those of the publisher, the editors and the reviewers. Any product that may be evaluated in this article, or claim that may be made by its manufacturer, is not guaranteed or endorsed by the publisher.

## Footnotes

1
http://huttenhower.sph.harvard.edu/kneaddata

2
https://www.ncbi.nlm.nih.gov/refseq/

3
https://www.uniprot.org/

4
https://metacyc.org/

5
https://omics.informatics.indiana.edu/MinPath/

## Supplementary Material

Supplementary Figure 1 Alpha diversity dilution curves. Each group contained six samples. The abscissa is the total number of sequences, while the ordinate represents the number of OTUs. **(A)** Healthy and disease rhizosphere soil chao1 alpha diversity dilution curves. **(B)** Healthy and disease rhizosphere soil observed alpha diversity dilution curves. **(C)** Healthy and disease root endophyte chao1 alpha diversity dilution curves. **(D)** Healthy and disease root endophyte observed alpha diversity dilution curves.

Supplementary Figure 2 Boxplots of diversity in the rhizosphere soil and root endophyte group. **(A)** ACE index box plot based on the rhizosphere soil and root endophyte bacteria species abundance. **(B)** Chao1 index box plot based on the rhizosphere soil and root endophyte bacteria species abundance. **(C)** Invsimpson index box plot based on the rhizosphere soil and root endophyte bacteria species abundance. **(D)** Simpson index box plot based on the rhizosphere soil and root endophyte bacteria species abundance.

Supplementary Figure 3 Annotation results of bacteria with significantly different functional descriptions. **(A)** Amino acid transport and metabolism, cell motility, posttranslational modification, protein turnover, and chaperones, and unknown function. **(B)** Antibiotic gene percentage of each group. **(C)** Venn diagram of KEGG orthologs (KO) functional annotation results for each group.

Supplementary Figure 4 Quantitative heat map of the bins in each group. **(A)** Quantitative heat map of the bins in healthy rhizosphere soil. **(B)** Quantitative heat map of the bins in disease rhizosphere soil. **(C)** Quantitative heat map of the bins in healthy root endophyte. **(D)** Quantitative heat map of the bins in disease root endophyte.

Supplementary Figure 5 Vitro infection results of *Pseudomonas fluorescens*.

Supplementary Table 1 Sample and data information.

Supplementary Table 2 Eukaryote information for each sample.

Supplementary Table 3 Function annotation information.

Supplementary Table 4 Genomic and classification information of the bins in each group.
