# Supplementary figures and images for: Structure and Function of Rhizosphere Soil and Root Endophytic Microbial Communities Associated With Root Rot of Panax notoginseng
Source: Front Plant Sci. 2022 Jan 5;12:752683. doi: 10.3389/fpls.2021.752683 (PMC8766989; doi:10.3389/fpls.2021.752683)

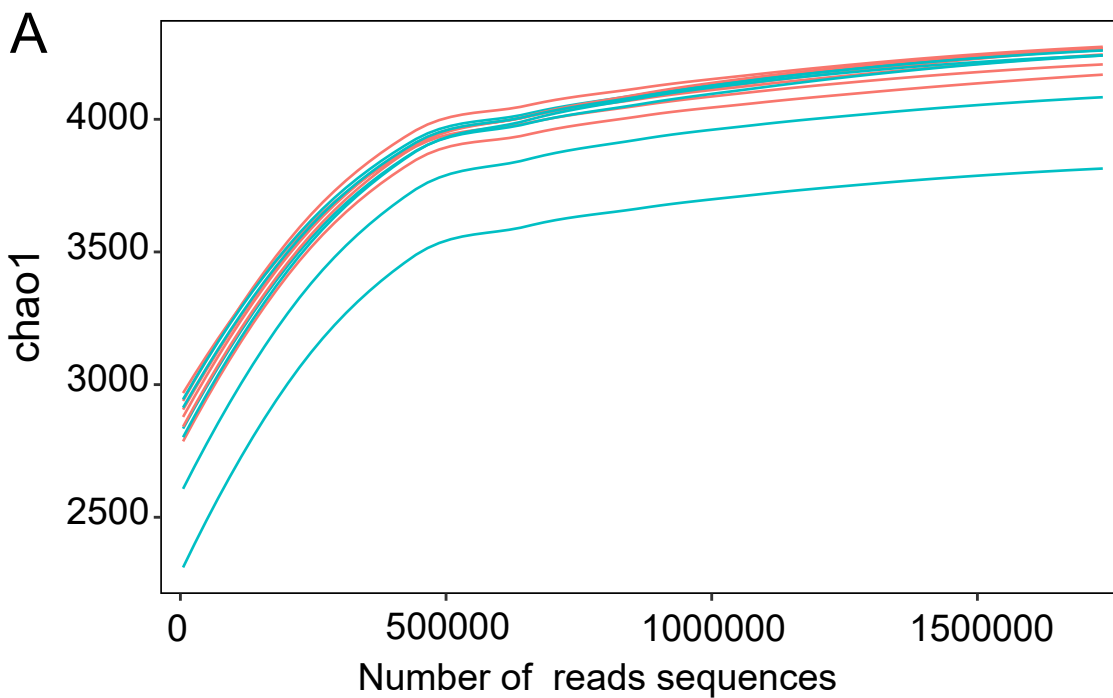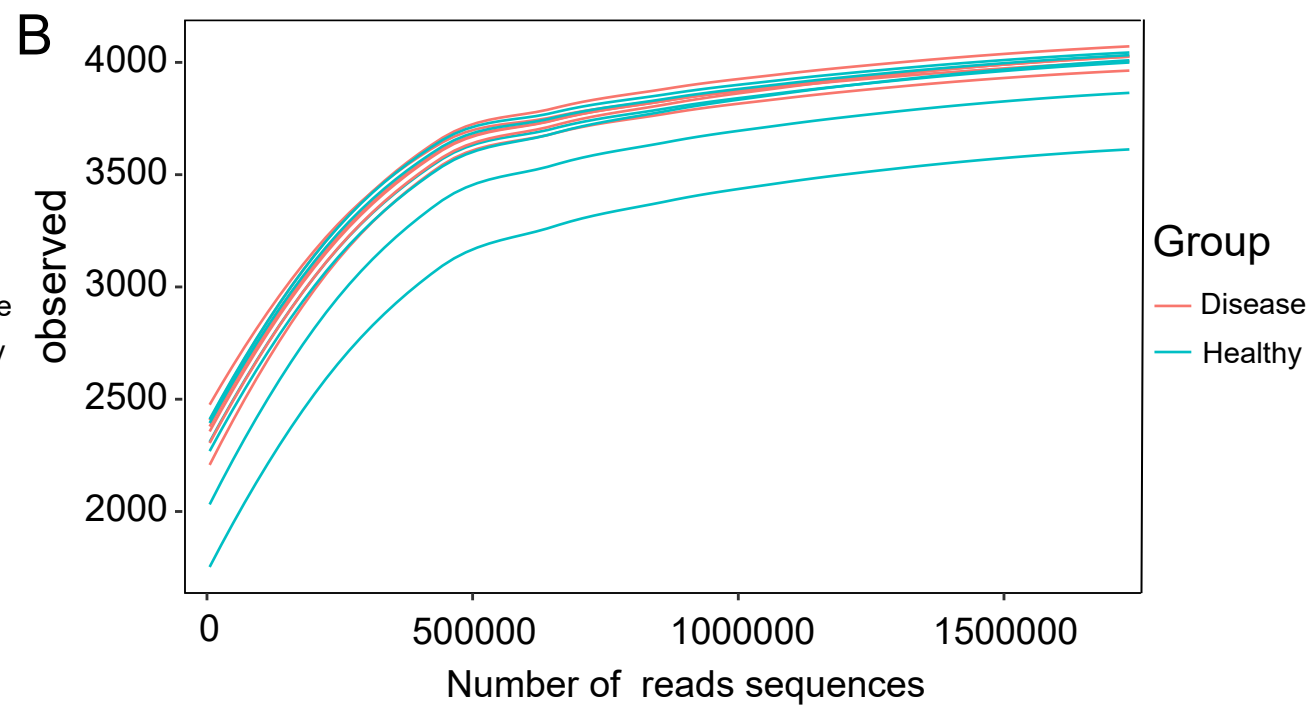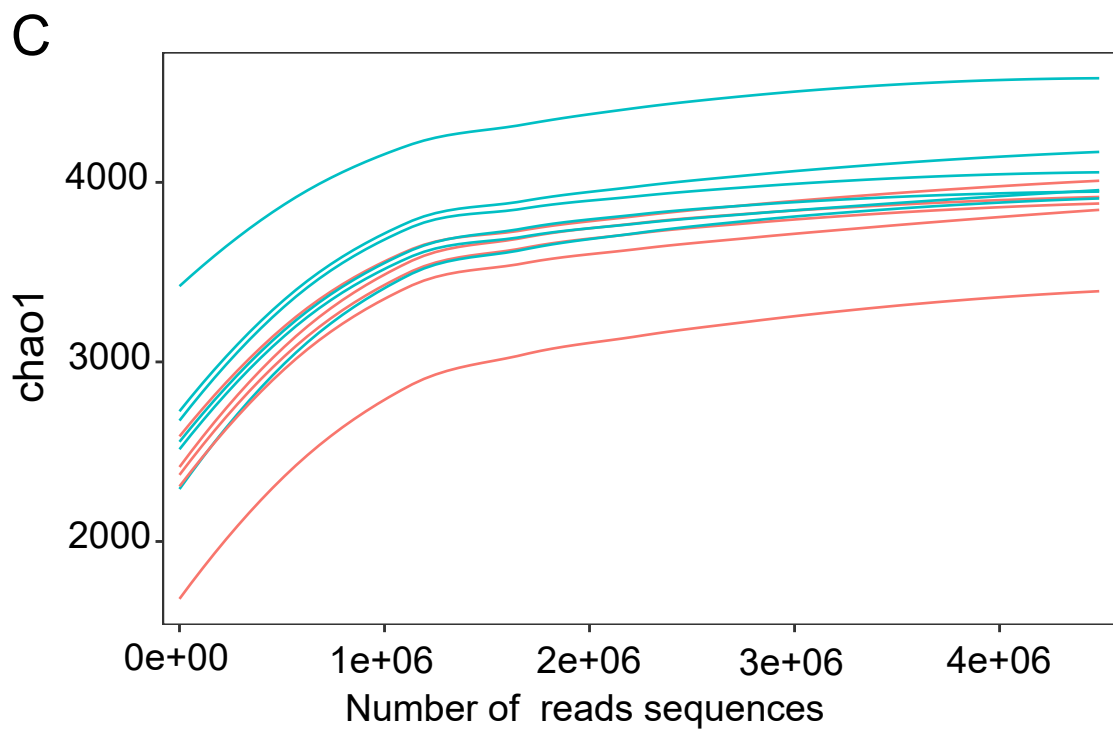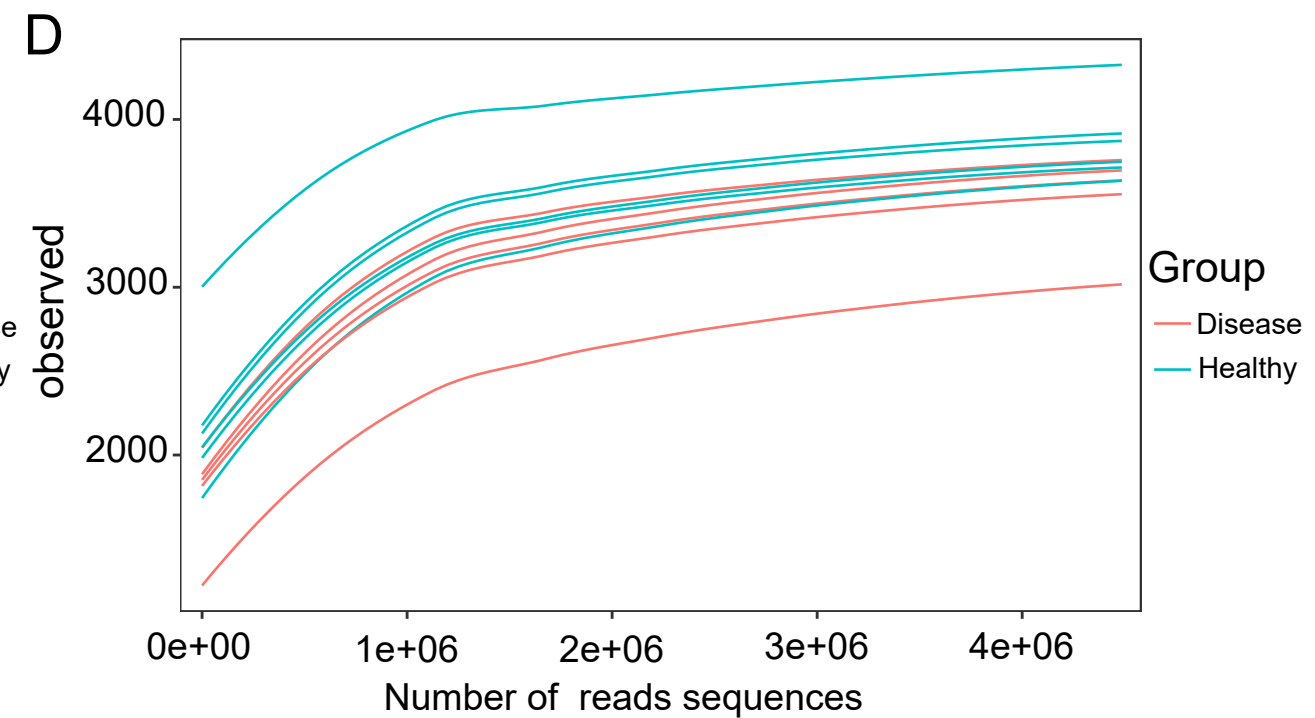

Supplement: Supplementary Figure 1 — Alpha diversity dilution curves. Each group contained six samples. The abscissa is the total number of sequences, while the ordinate represents the number of OTUs. (A) Healthy and disease rhizosphere soil chao1 alpha diversity dilution curves. (B) Healthy and disease rhizosphere soil observed alpha diversity dilution curves. (C) Healthy and disease root endophyte chao1 alpha diversity dilution curves. (D) Healthy and disease root endophyte observed alpha diversity dilution curves. [file Image_1.pdf]

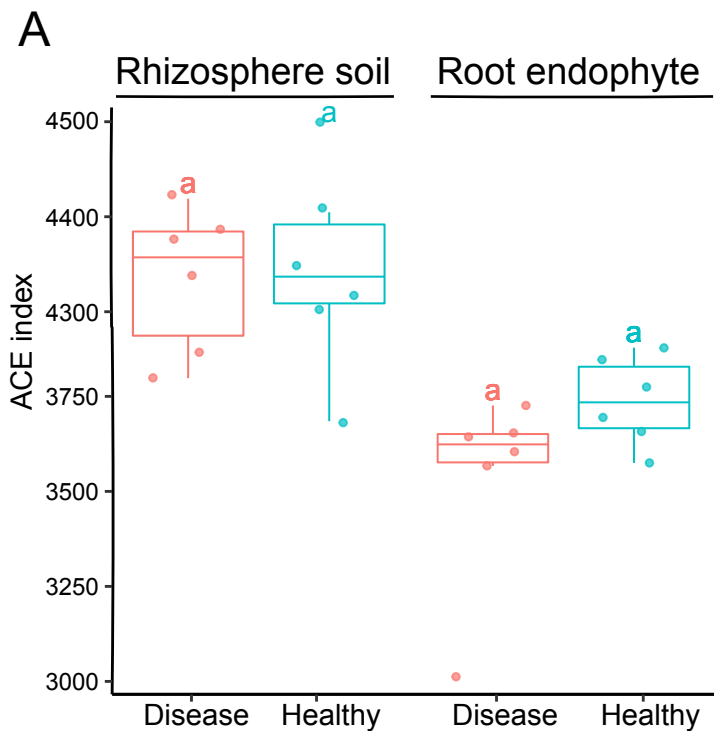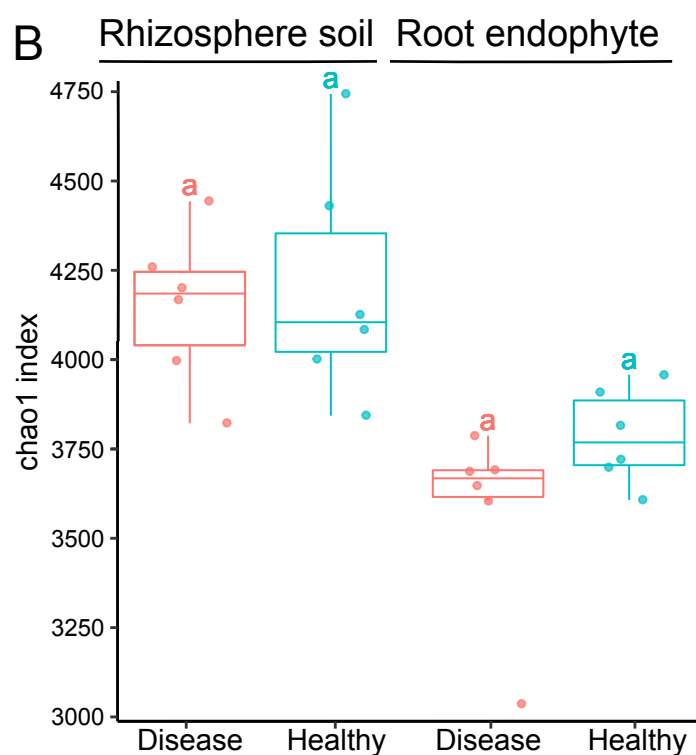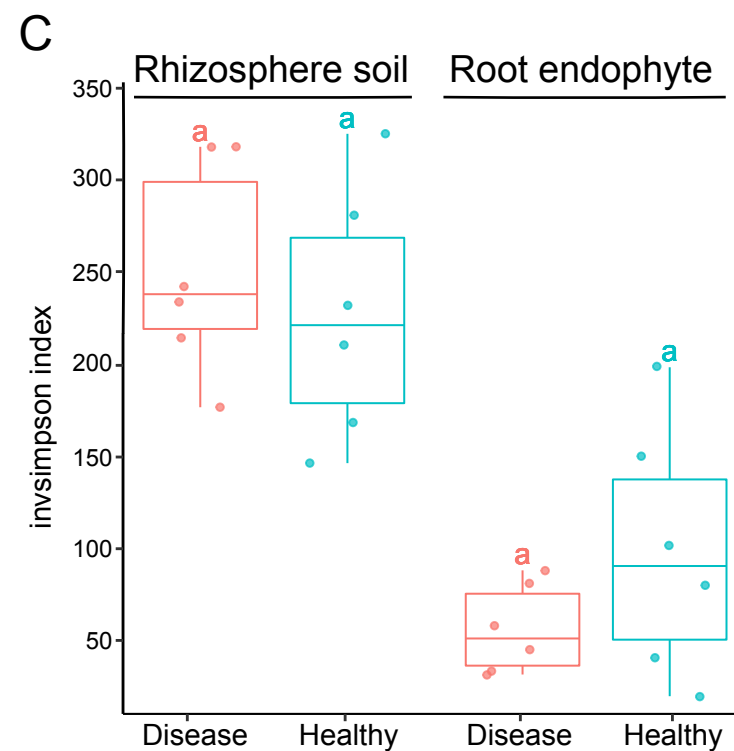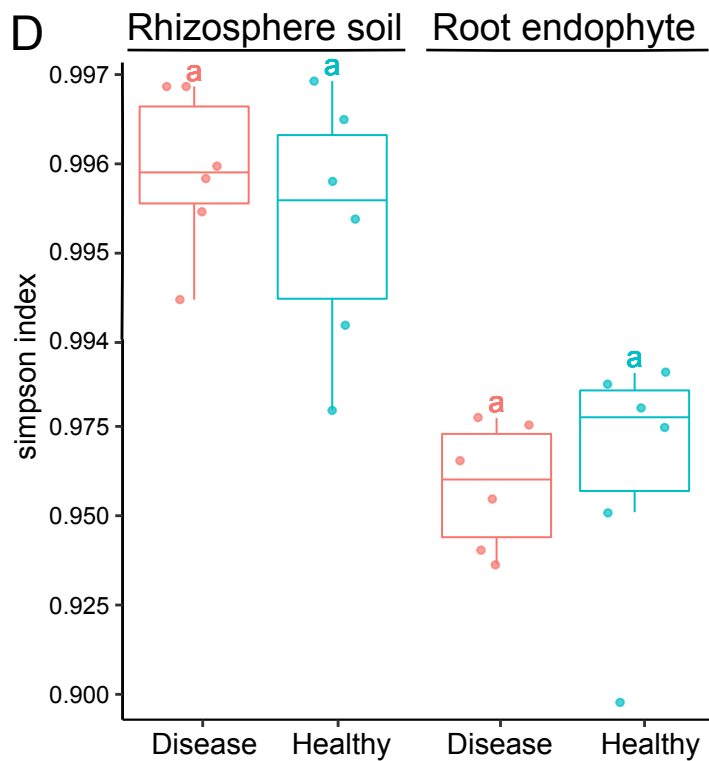

Supplement: Supplementary Figure 2 — Boxplots of diversity in the rhizosphere soil and root endophyte group. (A) ACE index box plot based on the rhizosphere soil and root endophyte bacteria species abundance. (B) Chao1 index box plot based on the rhizosphere soil and root endophyte bacteria species abundance. (C) Invsimpson index box plot based on the rhizosphere soil and root endophyte bacteria species abundance. (D) Simpson index box plot based on the rhizosphere soil and root endophyte bacteria species abundance. [file Image_2.pdf]

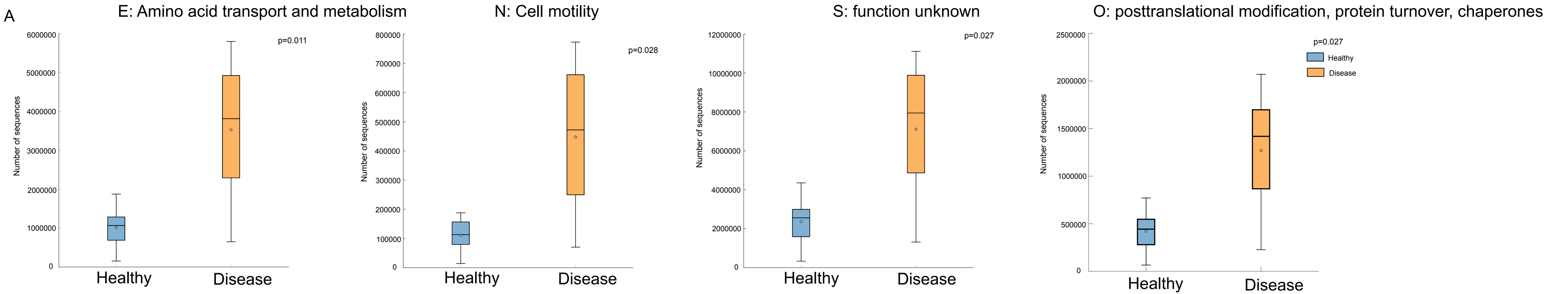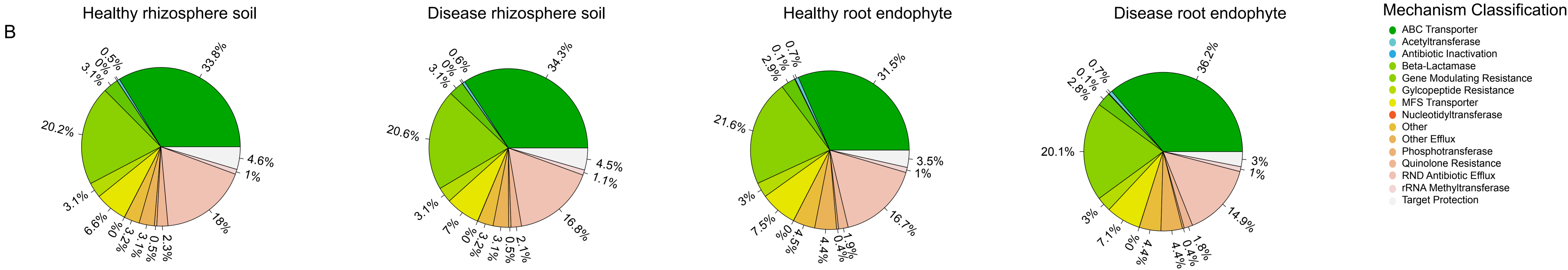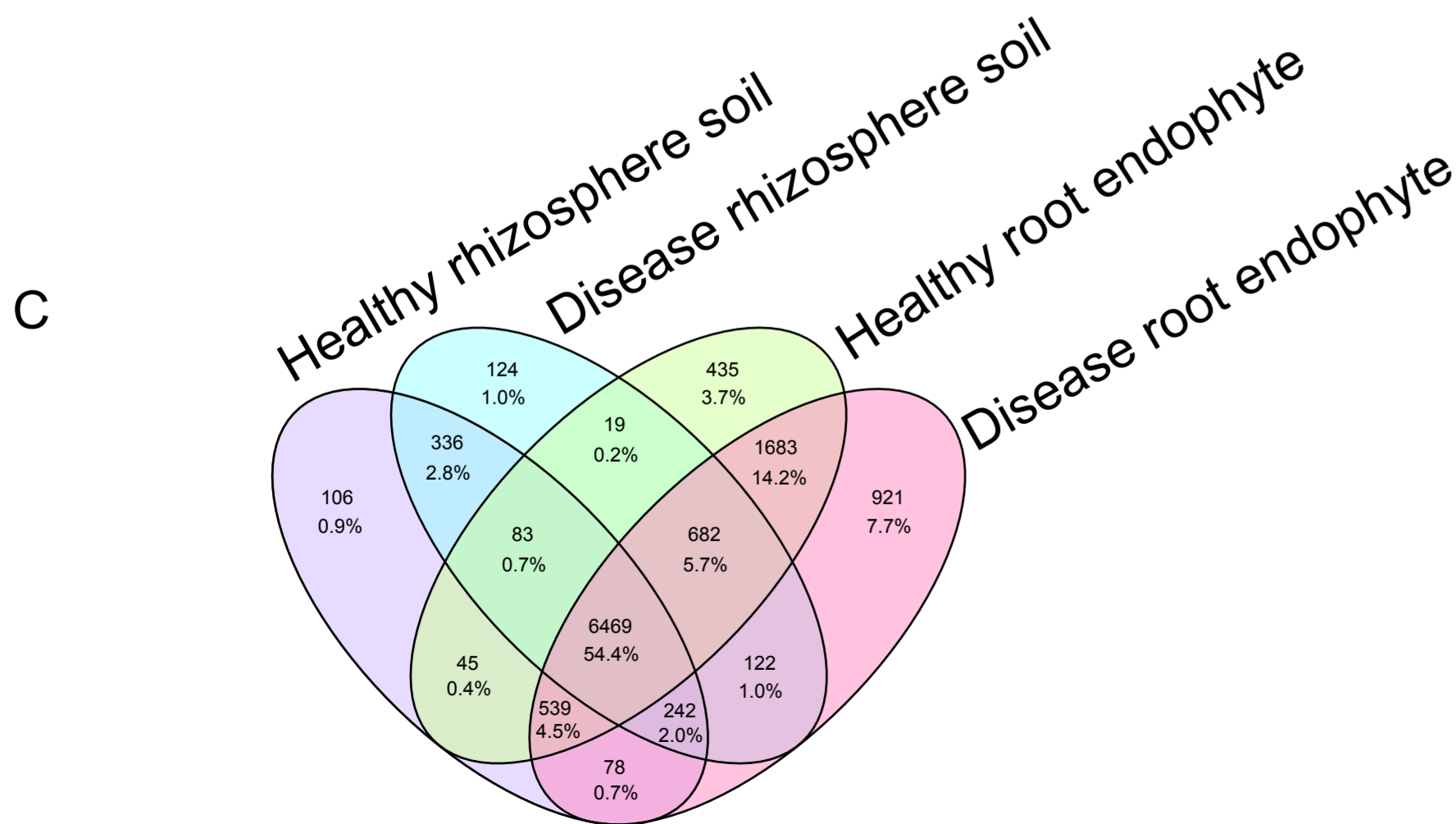

Supplement: Supplementary Figure 3 — Annotation results of bacteria with significantly different functional descriptions. (A) Amino acid transport and metabolism, cell motility, posttranslational modification, protein turnover, and chaperones, and unknown function. (B) Antibiotic gene percentage of each group. (C) Venn diagram of KEGG orthologs (KO) functional annotation results for each group. [file Image_3.pdf]

A

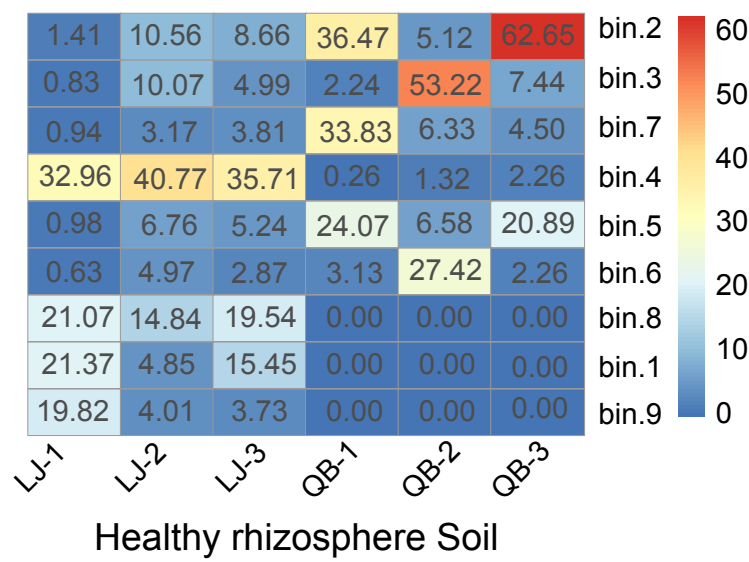

B

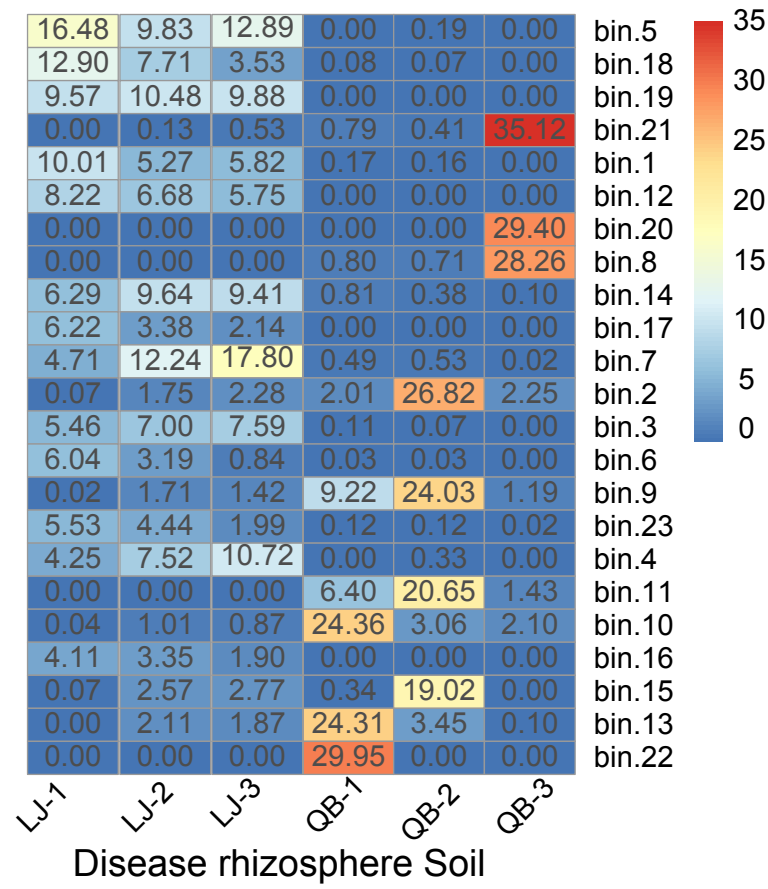

C

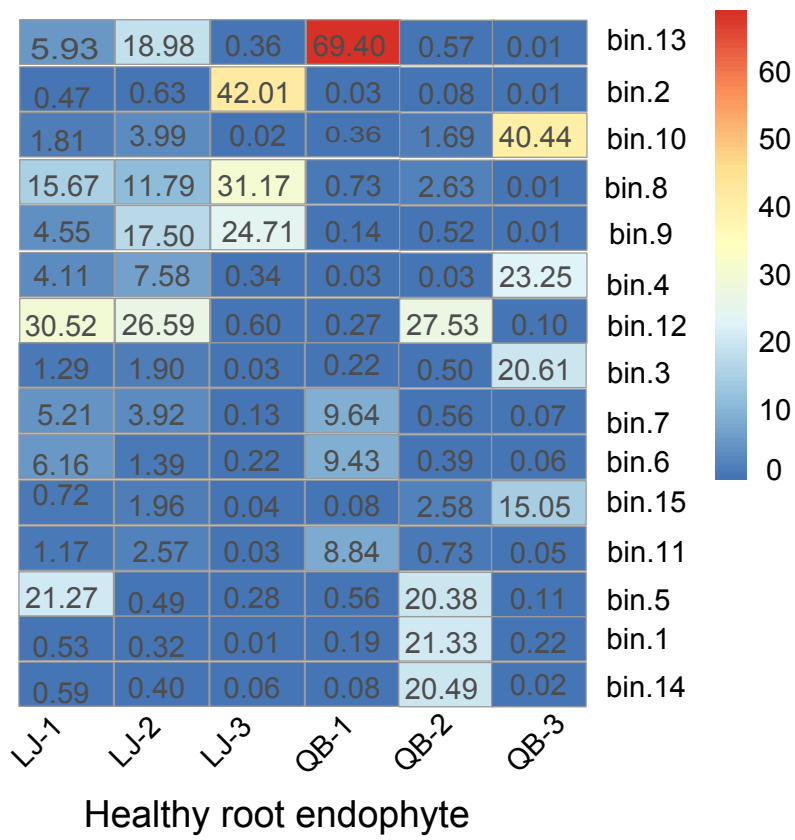

D

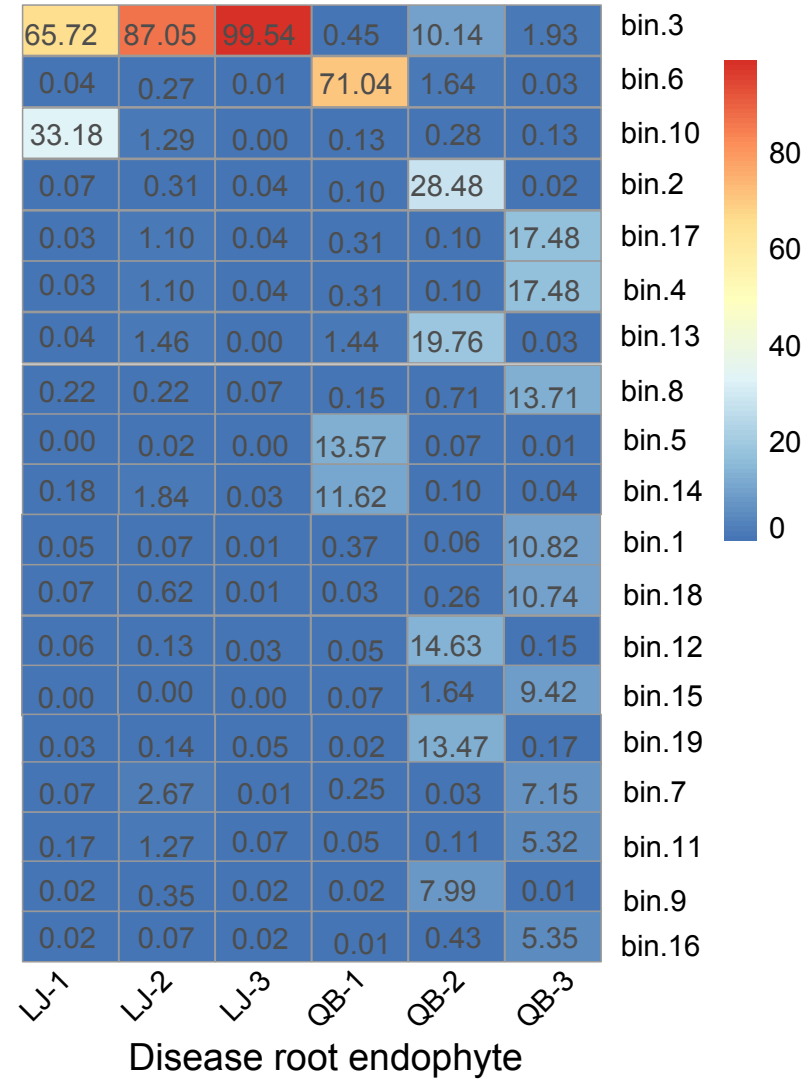

Supplement: Supplementary Figure 4 — Quantitative heat map of the bins in each group. (A) Quantitative heat map of the bins in healthy rhizosphere soil. (B) Quantitative heat map of the bins in disease rhizosphere soil. (C) Quantitative heat map of the bins in healthy root endophyte. (D) Quantitative heat map of the bins in disease root endophyte. [file Image_4.pdf]

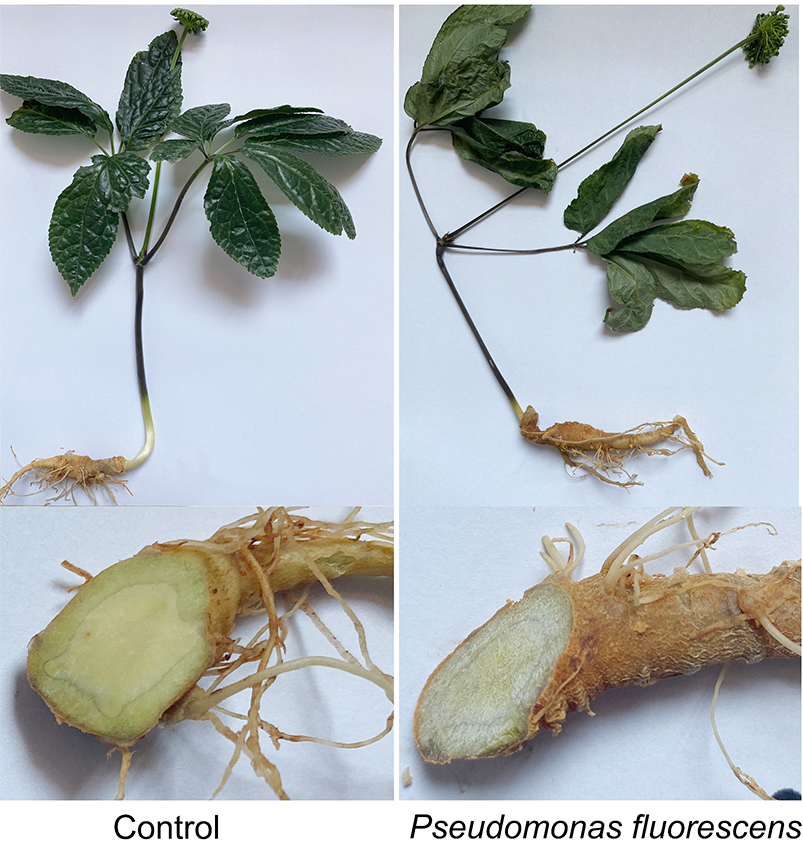

Supplement: Supplementary Figure 5 — Vitro infection results of Pseudomonas fluorescens. [file Image_5.JPEG]
